# Tracheitis; Condensation of the Lower Third of the Left Lung; Death; Inspection

**Published:** 1846-05

**Authors:** C. M. Durrant


*Tracheitis; Condensation of the Lower Third of the Left Lung; Death; Inspection.* By C. M. Durrant, M. D.—A little girl, aged 4, after exposure to cold, was seized with catarrh and hoarseness, which was permitted to continue for five days prior to seeking medical advice.

On the fifth day the symptoms were those of croup, with stridulous breathing and cough; at which period she was freely leeched over the sternum, had repeated emetics, and took calomel and antimony every two hours; blisters were applied to the nape and sides of the neck. I saw her in conjunction with her medical attendant on the eleventh day of the disease. Her complexion was pale; skin hot and pungent; tongue tolerably clean; the bowels had been freely relieved; urine depositing lithates; pulse quick and feeble; breathing accelerated and slightly stridulous; cough short, not very frequent, and unaccompanied by expectoration. A minute physical examination of the chest could not be made. Percussion elicited marked dulness over the postero-inferior regions of both sides, while auscultation afforded evidence of the lungs being very imperfectly filled with air.

The previous treatment, viz., two grains of calomel, with half a grain of tartar emetic, every two hours, was persevered in; the trachea was blistered by means of the application of a roll of flannel dipped in boiling water; and the vapour of water was directed to be kept constantly diffused throughout the air of the room.

The little patient, although decidedly, was but temporarily, relieved by these measures; she became rapidly weaker, in spite of stimulants, and died comatose on the thirteenth day of the disease.

*Inspection.* Mucous membrane of the larynx and trachea intensely red, and dry; three small abraded spots, the result of commencing ulceration, were found upon the mucous membrane of the trachea, immediately above its bifurcation; bronchial tubes injected and filled with sanguineous mucus; right lung healthy; liver protruding high into the right pleura, giving the character of dulness on percussion during life; lower fourth of left lung hepatized; the apex of the same lung contained two or three scattered, but softened tubercles; left costal pleura covered with recently effused albuminous lymph.

*Remarks.* To the length of time which was so culpably allowed to intervene between the commencement of the disease in the case just detailed, and the period at which medical advice was sought, is mainly to be attributed the want of success attending the very energetic and judicious treatment in the first instance adopted.

The general symptoms were those of inflammation of the trachea, with obstructed respiration, and do not call for special comment. The dulness on percussion over the right infra-scapular region was more marked during life than upon the left side, and affords another instance of protrusion of the liver, simulating disease within the chest. With the exception of the dulness over the lower part of the lungs behind, the natural resonance on percussion was unaffected. Auscultation, on the contrary, afforded evidence of extreme feebleness of the respiratory murmur, a degree of spasm of the commencement of the air passages preventing free access to the entrance of air.

Spasm of the glottis, in croup, rather than the amount of adventitious membrane, becomes very frequently the immediate cause of death ; to obviate which, (as was given in the above case, although omitted in the report,) it is desirable to combine with the other remedies, small doses of an opiate. Death in the above case occurred probably in part from the shock of the nervous system, by the extent of the inflammation and duration of the disease, and partly also from the influence exerted on the brain by the circulation of imperfectly arterialized blood.

The application of boiling water for the purpose of exciting rapid vesication will, in many cases, similar to the one in question, be found of signal benefit. The treatment of disease by moist air, and the influence which it exerts in depurating internal organs when in a state of congestion, has been ably revived by Dr. Golding Bird. The diseases to which this practice is more especially applicable are, capillary bronchitis, pneumonia, and croup in children; its adoption may, however, be extended with great propriety and advantage to similar affections in the adult. Under its operation, as stated by Dr. Bird, and which I have also verified, the respiration becomes less rapid, and panting ; the cough diminishes frequently in a marked degree ; the secretion from the bronchi is rendered less viscid, and expectoration consequently facilitated, and diaphoresis occurs.

In two instances which have recently fallen under my notice, the most beneficial effects followed the diffusion of moist air as an auxiliary to the ordinary treatment.

The first case was that of an infant, aged eighteen months, in whom the most intense capillary and general bronchitis existed. The temperature of the room was steadily maintained at 70° Fahrenheit, while a constant supply of steam was conveyed through a tube into the apartment, both during the day and night. After the expiration of five hours a marked improvement in the symptoms became visible, the respirations less frequent, with diminution of cough and frequency of pulse, and the ultimate recovery was rapid.

The second case was bronchitis, supervening on latent pneumonia, occurring in a young gentleman, aged 15. The posterior aspect of the left lung, embracing the infra-scapular and lateral regions, was consolidated, over the entire extent of which the respiration was so loudly tubular, as to resemble cavernous breathing. The cough, which was the most distressing symptom, was nearly constant. In this case the diffusion of moist air, unremittingly supplied, was equally efficacious as in the former, in lessening the irritation, and finally removing the cough.—*Lon. Med. and Sur. Jour.*

